# Chronic subordination stress selectively downregulates the insulin signaling pathway in liver and skeletal muscle but not in adipose tissue of male mice

**DOI:** 10.3109/10253890.2016.1151491

**Published:** 2016-03-07

**Authors:** Valentina Sanghez, Cankut Cubuk, Patricia Sebastián-Leon, Stefania Carobbio, Joaquin Dopazo, Antonio Vidal-Puig, Alessandro Bartolomucci

**Affiliations:** ^a^ Department of Integrative Biology and Physiology, University of Minnesota, Minneapolis, MN, USA; ^b^ Department of Neuroscience, University of Parma, Parma, Italy; ^c^ Department of Computational Genomics, Centro de Investigación Principe Felipe, Valencia, Spain; ^d^ Wellcome Trust MRC Metabolic Disease Unit, Institute Metabolic Science, Addenbrooke’s Hospital, University of Cambridge, Cambridge, UK; ^e^ Wellcome Trust Sanger Institute, Hinxton, UK

**Keywords:** Adipose tissue, insulin, IRS1, IRS2, metabolic syndrome, obesity

## Abstract

Chronic stress has been associated with obesity, glucose intolerance, and insulin resistance. We developed a model of chronic psychosocial stress (CPS) in which subordinate mice are vulnerable to obesity and the metabolic-like syndrome while dominant mice exhibit a healthy metabolic phenotype. Here we tested the hypothesis that the metabolic difference between subordinate and dominant mice is associated with changes in functional pathways relevant for insulin sensitivity, glucose and lipid homeostasis. Male mice were exposed to CPS for four weeks and fed either a standard diet or a high-fat diet (HFD). We first measured, by real-time PCR candidate genes, in the liver, skeletal muscle, and the perigonadal white adipose tissue (pWAT). Subsequently, we used a probabilistic analysis approach to analyze different ways in which signals can be transmitted across the pathways in each tissue. Results showed that subordinate mice displayed a drastic downregulation of the insulin pathway in liver and muscle, indicative of insulin resistance, already on standard diet. Conversely, pWAT showed molecular changes suggestive of facilitated fat deposition in an otherwise insulin-sensitive tissue. The molecular changes in subordinate mice fed a standard diet were greater compared to HFD-fed controls. Finally, dominant mice maintained a substantially normal metabolic and molecular phenotype even when fed a HFD. Overall, our data demonstrate that subordination stress is a potent stimulus for the downregulation of the insulin signaling pathway in liver and muscle and a major risk factor for the development of obesity, insulin resistance, and type 2 diabetes mellitus.

## Introduction

Obesity and type 2 diabetes mellitus (T2D) are characterized by a multifactorial and polygenic etiology (Bouchard, 1991; Kahn, 1994; Stumvoll et al., 2005). Failure of chronic stress adaptation and socioeconomical challenges have been related to neuroendocrine and autonomic dysregulations leading to visceral obesity, increase in body mass index (BMI), and development of the metabolic syndrome (MetS) and insulin resistance (Mackenbach et al., 1997; Rosmond et al., 1998; van Strien et al., 1986). Supportive evidence is provided for the positive association between hypercortisolemia, increased body weight, and T2D (Kyrou et al., 2006; Shpilberg et al., 2012). Chronic activation of stress response systems (Koolhaas et al., 2011; Sapolsky et al., 2000) is characterized by visceral fat accumulation and insulin resistance (Kyrou et al., 2006) associated with high release of glucocorticoids (Dallman et al., 2006). Moreover, elevated glucocorticoid concentrations increase hepatic gluconeogenesis, increase plasma glucose concentration, and impair the anabolic action of insulin (Dallman, 2010; Dallman et al., 1993; Sapolsky et al., 2000). However, increased plasma corticosterone concentration, which is consistently a feature of chronic stress models, is not always associated with obesity vulnerability. Indeed, several models of stress are associated with hypophagia or unaltered food intake and weight loss (Dallman et al., 2006; Harris, 2015; Razzoli et al., 2016). Despite several genetic or pharmacological animal models of MetS and T2D having been developed, so far there is a paucity of models linking socioeconomic status, stress, MetS, and T2D that are suitable to investigate the underlying molecular mechanisms. Social subordination stress has long been considered ideal to mimic the impact of psychosocial stress on human pathologies (Bartolomucci et al., 2005; Koolhaas et al., 2011; Sapolsky, 2005; Scott et al., 2012). We previously validated a mouse model of chronic psychosocial stress (CPS)-induced derangements toward obesity and MetS (Bartolomucci et al., 2005,2009; Dadomo et al., 2011; Sanghez et al., 2013). Based on these results, we hypothesized that this phenotype may provide key information about early pathogenically relevant metabolic disturbances induced by psychosocial stress without the confounding secondary effects derived from severe metabolic disturbance in morbidly obese animals. Specifically, we tested the hypothesis that the metabolic difference between subordinate and dominant mice was associated with changes in functional pathways relevant for insulin sensitivity, glucose and lipid homeostasis. Accordingly, we combined the analysis of candidate metabolic genes regulating insulin signaling, glucose and lipid homeostasis by real-time quantitative PCR in liver, skeletal muscle (quadriceps), and perigonadal white adipose tissue (pWAT; pWAT was selected because it is one of the largest and better characterized visceral fat pads in mice (de Jong et al., 2015)) of subordinate and dominant mice fed a standard diet, and for comparison in control mice fed a high-fat diet (HFD). Further we analyzed by qPCR the same genes in mice undergoing stress and fed a HFD. Finally, we used a novel bioinformatic analysis of selected functional signaling pathways (insulin, peroxisome proliferator-activated receptor (PPAR), and adipokines).

## Methods

### Animals

Male CD1 mice derived from an outbred stock were obtained from Charles River, Lecco, Italy. Mice were reared in groups of 4–6 same sex siblings in a 12:12 h light:dark cycle (lights on at 07:00 h) at 22 ± 2 °C (see Razzoli et al., 2016, for a discussion on the relevance of the environmental temperature on the stress-induced metabolic phenotype). Animal experiments were conducted at the University of Parma (Italy) and approved by the ethical committee of the University of Parma.

Diets: Mice were fed standard diet (4RF21, Mucedola, Milano, Italy; 3.9 Kcal/g, 6.5% Kcal from fat) or HFD (Mucedola modified 4RF21, 5.2 Kcal/g, and 45% Kcal from fat).

### Chronic psychosocial stress protocol

We used our standard protocol (Bartolomucci et al., 2004, 2005, 2009; Dadomo et al., 2011; Sanghez et al., 2013) in which stable resident/intruder pairs of adult male mice were formed after a baseline period lasting 5 d in which mice were isolated to establish territorial ownership and to collect basal metabolic parameters. At the beginning of the phase of stress for four weeks each resident received an unfamiliar weight-matched intruder mouse and the two mice were allowed to freely interact for 10 min. After the interaction, residents and intruders were separated by a perforated partition, which allowed continuous visual, auditory, and olfactory sensory contact but no physical interaction. The partition was removed daily (between 08:00 h and 09:00 h), for a maximum of 10 min. During social interaction, offensive behavior was manually recorded and social status of the mice was determined. Only dyads that reliably showed a stable dominant/subordinate hierarchy and in which the subordinate showed no attack after day 4 were included in the study. Age and weight-matched mice, housed in groups of three siblings, were included as a control group according to our standard validated protocol (Bartolomucci et al., 2004; additional details are in Bartolomucci et al., 2004 and Sanghez et al., 2013). The metabolic phenotype of CPS-exposed mice has been described in detail (Sanghez et al., 2013); previously published physiological results are presented in Supplementary Table S1 for reference. The present study investigated the molecular changes in tissues collected from the same mice described in our previous paper (Sanghez et al., 2013). Mice were euthanized by decapitation following brief CO_2_ exposure between 09:00 h and 11:00 h within 3 min after an experimenter entered the animal room. Liver, quadriceps (hereafter referred to as skeletal muscle), and pWAT were collected under aseptic conditions and stored in RNase-free tubes at −80 °C.

### Real-time PCR

Total RNA was isolated from pWAT, liver, and skeletal muscle using STAT60 isolation reagent (Tel-Test, Inc., Friendswood, TX). RNA integrity was assessed with electrophoresis agarose gel by Sybr-safe stain (Invitrogen, Carlsbad, CA). Real-time quantitative PCR was performed to quantify the expression of candidate genes involved in insulin signaling, glucose and lipid metabolism (a full list of genes measured including values for genes showing no significant changes in expression are in Supplementary Table S2), using TaqMan or a SybrGreen sequence detection system on an ABI 7900 instrument (Applied Biosystem, Foster City, CA) as described by (Pfaffl et al., 2004). Expression of each target gene was corrected by the geometrical average of four different housekeeping genes: 18S, β2-microglobulin, β-actin, and 36B4 using the best-keeper tool (Pfaffl et al., 2004).

### Statistical analysis

Data were analyzed with unpaired *t*-tests, two-way ANOVA, and two-way repeated measures ANOVA followed by Tukey’s HSD or Duncan post hoc tests by using Statistica (Statsoft, Inc., Tulsa, OK). *p* values less than 0.05 were considered statistically significant. Significance level is indicated in the figure legends. Data are presented as mean + SEM.

### Pathway analysis

We used here a probabilistic approach to analyze the possible different functionalities resulting from the different ways in which signals can be transmitted across a pathway. Gene activity, estimated from the level of expression, can be used within a probabilistic context to calculate the probabilities of a signal to be transmitted from the input node (receptor proteins) to the output node (effector proteins) in a pathway. Differential activity in distinct input/output connections will result in different functional activities triggered by the pathway. Here we used a new approach in which, instead of analyzing the activity of the pathway as a whole, we rather analyze the possible different functionalities resulting from the different ways in which signals can be transmitted across the pathway. These different stimulus-response pathways we call sub-pathways.

The method we used performs four steps. The first step is the modeling of signaling pathways extracted from KEGG (Kyoto Encyclopedia of Genes and Genomes). This is done once for each studied pathway by taking into account the relationships of activation or repression established between gene products. The second step consists of computing the activity of each gene product (calculated from the PCR expression experiment described earlier) in the modeled pathways. The normalized gene expression data are rescaled from the range of variation to a 0–1 interval range. As a result, the higher values represent the most expressed (or activated) data. Furthermore, a KEGG pathway node can contain one or more gene products. The node information is summarized using the 95% percentile of the corresponding normalized gene expression values. The method proposed to model the pathways can easily deal with missing data. The final score for relevant sub-pathways for which critical nodes are measured are calculated as weighted products of the normalized expression values. Therefore, a missing measurement can be substituted by a one in all the compared conditions (Hernansaiz-Ballesteros et al., 2015). Consequently, the contribution of this particular gene to the final score is null and only the contributions of the genes measured are taken into account. The third step is to calculate the probability of activation of each sub-pathway from a pathway, based upon the following concepts: (i) Input node is any receptor node which does not receive signal from any other node in the pathway and starts the signaling process according to the KEGG diagram, unless this node is an inhibitor; (ii) Output node is any effector node at the end of the transmission of the signal; (iii) Sub-pathway is a sequence of nodes between an input and a connected output node. The probabilities of each node along the sub-pathway are propagated using the “Inclusion–exclusion principle”. The propagated product of probabilities takes only into account the effect of the available gene measurements. Genes with no measurements available are set to 1 and, consequently, do not affect to the resulting product. Finally, after the second and third steps have been performed on each sub-pathway from a pathway and also on each sample of the experiment, a Wilcoxon test is applied in order to assess the significance of the differential activations of each sub-pathway, which will account only for the effect attributable to the measured genes. Thus, the limitation of not having measurements for all the genes in the pathway is partly overcome by the pathway analysis strategy used. The methodology for sub-pathway analysis is explained in detail elsewhere (Sebastián-León et al., 2013, 2014). The current analysis only includes pathways in which more than six genes were directly measured. Other pathways and sub-pathways were excluded. Figures were generated using the CellMaps tool (http://cellmaps.babelomics.org/) in the Babelomics platform (Alonso et al., 2015).

## Results

### Opposite stress-induced metabolic consequences in dominant and subordinate mice

We previously reported the opposite metabolic phenotypes developed by subordinate and dominant mice exposed to CPS (Bartolomucci et al., 2009; Sanghez et al., 2013). Specifically, despite showing similar food intakes and plasma corticosterone concentrations, subordinate and dominant mice exhibited opposite changes in body weight and adiposity (Supplementary Table S1 summarizes findings described previously; Sanghez et al., 2013). Overall, subordinate mice showed a significant gain in body weight and pWAT mass, an obesogenic effect exacerbated by HFD when compared with control mice. On the contrary, dominant mice were resistant to diet-induced obesity. HFD and subordination stress were associated with increased circulating fasting total cholesterol, HDL and nonesterified fatty acid (NEFA) concentrations. The combination of chronic subordination stress and HFD exacerbated these metabolic abnormalities (Supplementary Table S1). After four weeks of stress no significant changes in basal plasma glucose concentration or glucose tolerance were detected in mice fed a standard diet. However, when subordinate mice were fed a HFD, they showed fasting hyperglycemia and glucose intolerance in the glucose tolerance test (GTT) as well as high homeostatic model assessment of insulin resistance (HOMA-IR) and low quantitative insulin sensitivity check index (QUICKI) when compared to all the other experimental groups (Supplementary Table S1).

### High-fat diet-induced molecular changes in control mice

**Figure 1.**
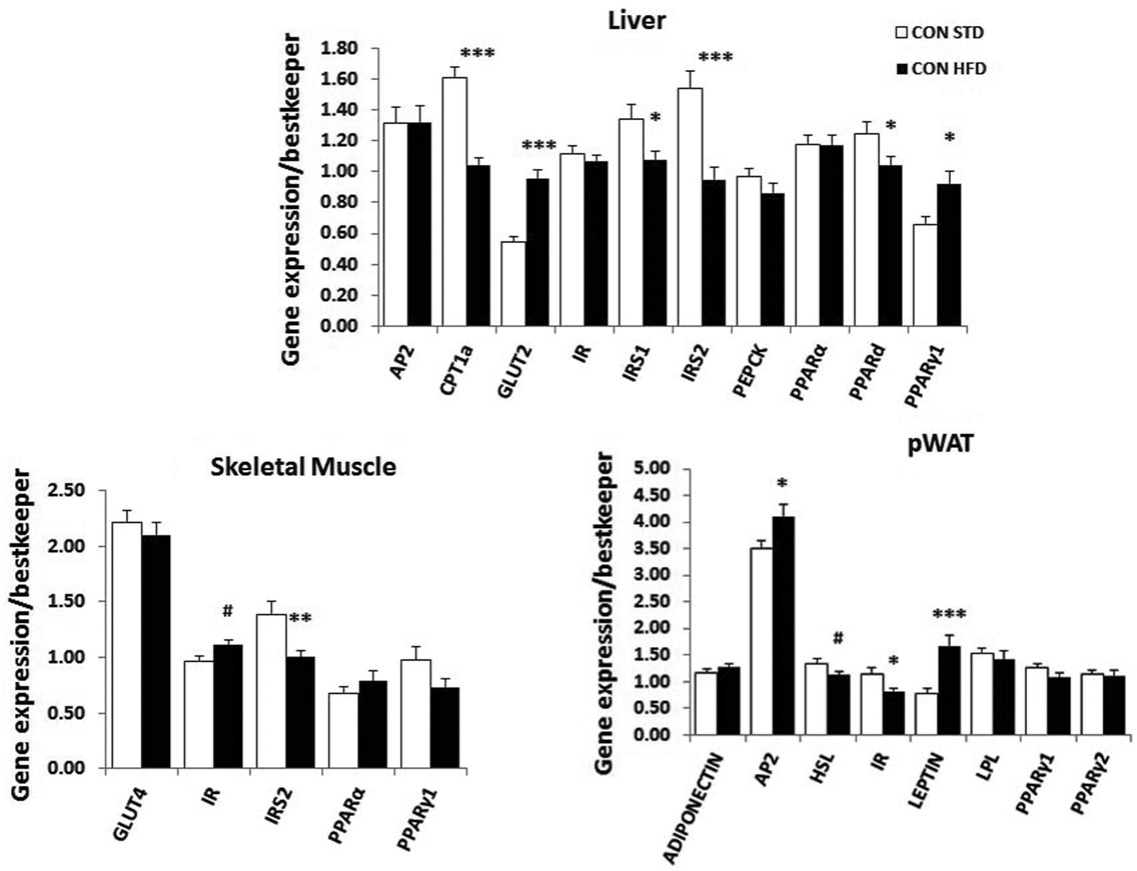
Molecular effect of HFD feeding in control mice. Real-time PCR analysis of candidate genes measured in the liver, muscle, and pWAT of control mice fed a standard (STD) or a high-fat diet (HFD). Data were analyzed using unpaired *t*-test, #*p* < 0.07 (tendency only), *p < 0.05, **p < 0.01, and ***p < 0.001. Number of mice per group (*n*): STD CON *n* = 16–19, HFD CON *n* = 20–23. Data are mean ± SEM.

As expected, the molecular analysis conducted in control mice-fed HFD for three weeks showed, compared to control mice-fed standard diet, that mRNA expression of candidate genes associated with insulin resistance and diabetes, such as insulin receptor substrates 1 and 2 (*IRS1, IRS2*), were downregulated in liver (*IRS1*, t_37 _=_ _2.5, *p* < 0.01; *IRS2*, t_37 _=_ _4.2, *p* < 0.001), while in skeletal muscle *IRS2* was downregulated (t_37 _=_ _3.1, *p* < 0.01). Moreover, carnitine palmitoyltransferase (*CPT1α*) mRNA was downregulated (t_37 _=_ _6.5, *p* < 0.0001), while the mRNA for insulin-independent glucose transporter *GLUT2* (t_36 _=_ _6.4, *p* < 0.0001) and insulin receptor (*IR*) (t_37 _=_ _3.0, *p* < 0.01) were upregulated in liver and skeletal muscle, respectively (Figure 1A). In pWAT, there was an increase in adipocyte protein 2 (*aP2*, t_36 _=_ _2.2, *p* < 0.05) and leptin (t_35 _=_ _3.8, *p* < 0.001) gene expression, while *PPARs* mRNA were not affected by HFD (Figure 1A).

### Opposite effect of social status on the molecular signature of MetS and insulin resistance in mice fed standard diet

**Figure 2.**
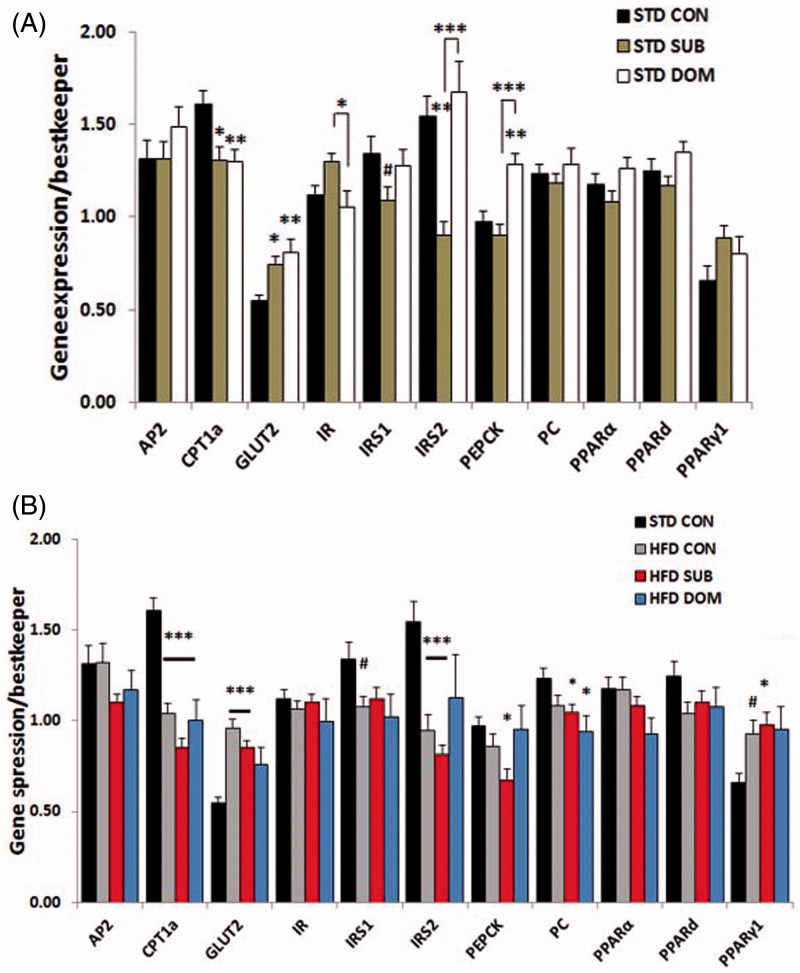
qPCR analysis of candidate genes in the liver of subordinate (SUB) and dominant (DOM) mice fed a (A) standard diet (STD) and (B) high-fat diet (HFD) compare to controls (CON). Data were analyzed using two-way ANOVA followed by Tukey’s HSD post hoc test, #*p* < 0.07, *p < 0.05,**p < 0.01, and ***p < 0.001. Asterisk (*) represents significant differences, hash sign (#) alone indicates tendency, vs. CON mice; bars identify significant differences between indicated treatments. Number of mice per group (*n*): STD CON *n* = 16–19, STD SUB *n* = 20–22, STD DOM *n* = 15–23, HFD CON *n* = 20–23, HFD SUB *n* = 22–26, and HFD DOM *n* = 7–8. Data are mean ± SEM. This figure will be best described with colors at the online version.

**Figure 3.**
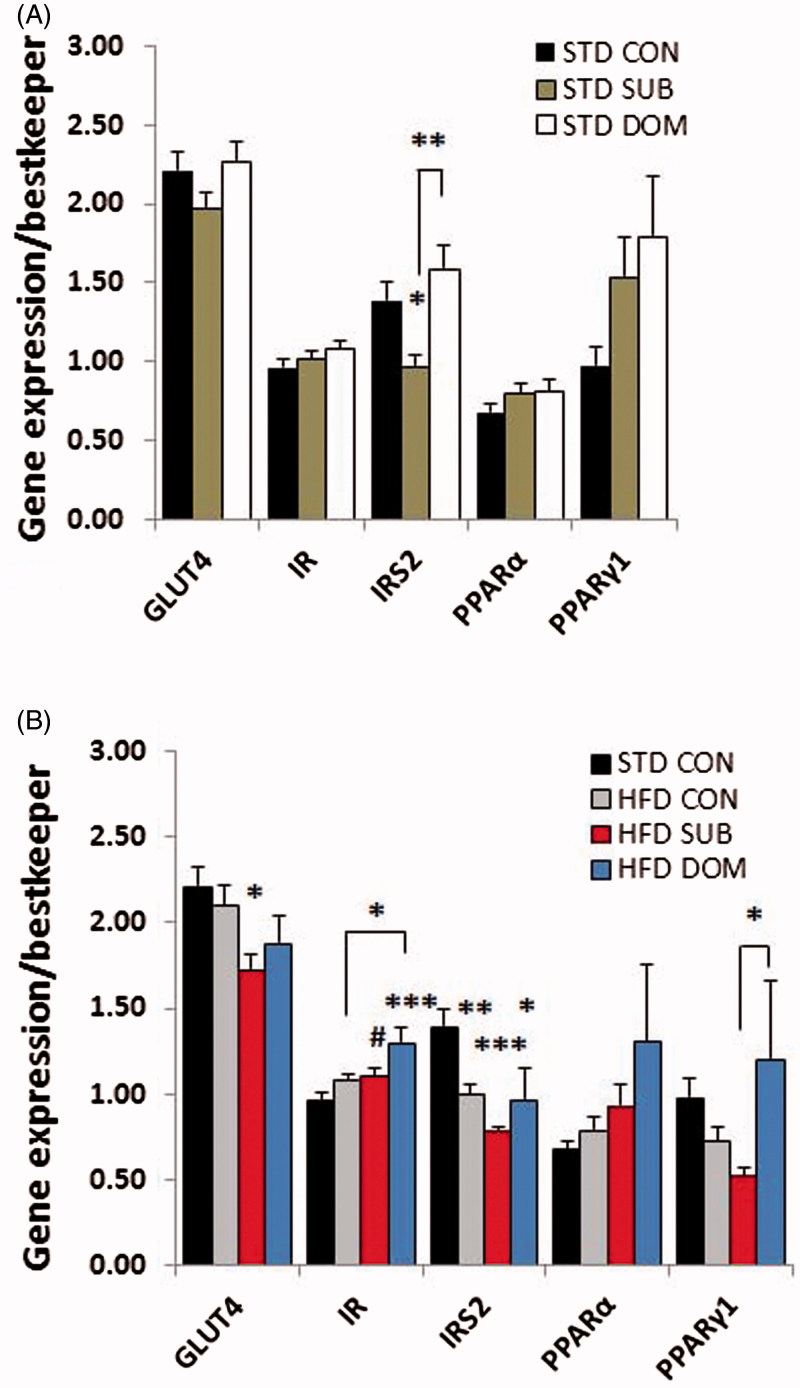
qPCR analysis of candidate genes in the skeletal muscle of subordinate (SUB) and dominant (DOM) mice fed a (A) standard diet (STD) and (B) high-fat diet (HFD) compare to Controls (CON). Data were analyzed using two-way ANOVA followed by Tukey’s HSD post hoc test. #*p* < 0.07, *p < 0.05,**p < 0.01, ***p < 0.001. Asterisk (*) represents significant differences, hash sign (#) alone indicates tendency, vs. CON mice; bars identify significant differences between indicated treatments. Number of mice per group (*n*): STD CON *n* = 16–19, STD SUB *n* = 20–22, STD DOM *n* = 15–23, HFD CON *n* = 20–23, HFD SUB *n* = 22–26, HFD DOM *n* = 7–8. Data are mean ± SEM. This figure will be best described with colors at the online version.

**Figure 4.**
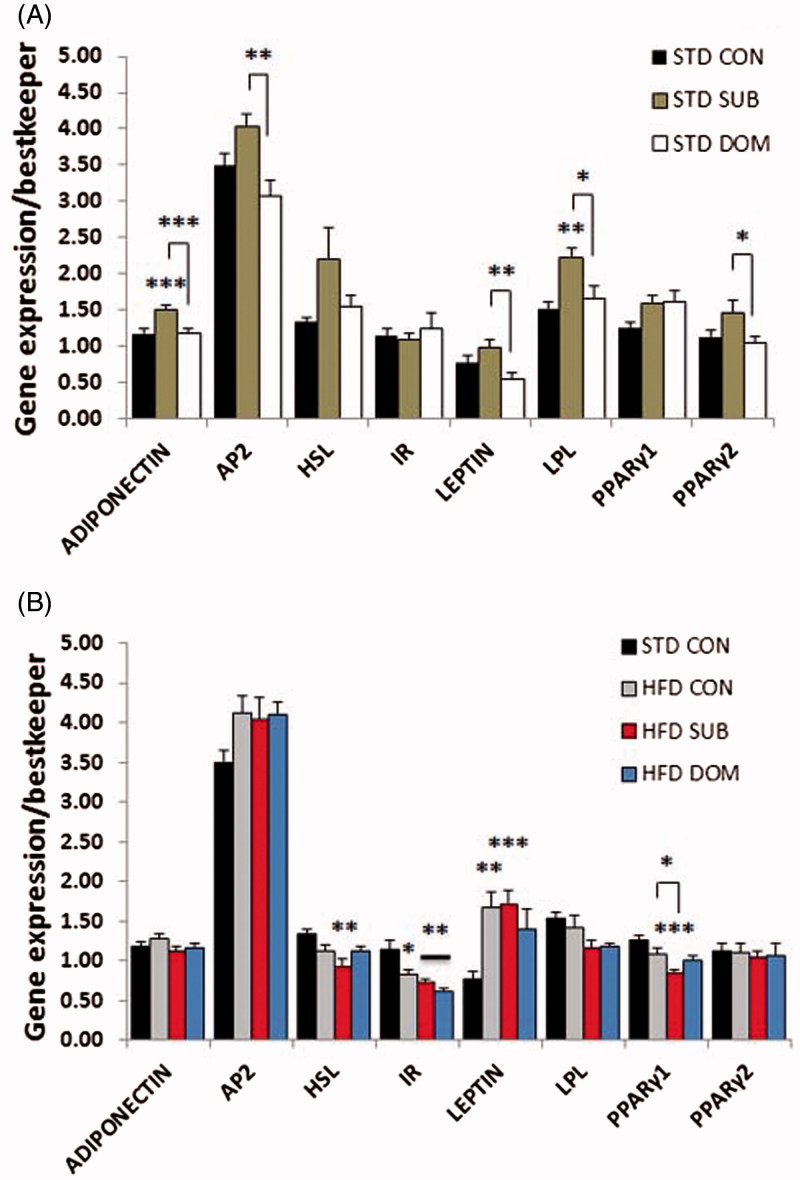
qPCR analysis of candidate genes in the pWAT of subordinate (SUB) and dominant (DOM) mice fed a (A) standard diet (STD) and (B) high-fat diet (HFD) compared to Controls (CON). Data were analyzed using two-way ANOVA followed by Tukey’s HSD post hoc test. #*p* < 0.07, *p < 0.05, **p < 0.01, and ***p < 0.001. Asterisk (*) represents significant differences, hash sign (#) alone indicates tendency, vs. CON mice; bars identify significant differences between indicated treatments. Number of mice per group (*n*): STD CON *n* = 16–19, STD SUB *n* = 20–22, STD DOM *n* = 15–23, HFD CON *n* = 20–23, HFD SUB *n* = 22–26, HFD DOM *n* = 7–8. Data are mean ± SEM. This figure will be best described with colors at the online version.

Our molecular analysis revealed that subordinate mice fed a standard diet, despite remaining euglycemic, exhibited molecular signatures of insulin resistance in liver and muscle, but not in pWAT (Supplementary Table S1). Analysis of the candidate genes with quantitative PCR showed in subordinate mice decreased *IRS2* and *CPT1α*, but not of *IRS1*, gene expression in liver (Figure 2; *IRS2, F*(2,52) = 11.6, *p* < 0.0001, *CPT1α, F*(2,52) = 6.24, *p* < 0.01), and of *IRS2* gene expression in skeletal muscle (Figure 3; *F*(2,49) = 7.1, *p* < 0.01), while expression of *GLUT2* and *IR* genes were upregulated in liver (Figure 3; *GLUT2, F*(2,52) = 6.3, *p* < 0.01, *IR, F*(2,52) = 3.9, *p* < 0.05). The pWAT (Figure 4) of subordinate mice showed an anabolic pattern of lipid metabolism gene expression characterized by increased expression of genes for lipoprotein lipase (*LPL, F*(2,52) = 7.44, *p* < 0.01), adipocyte protein 2 (*aP2, F*(2,53) = 7.5, *p* < 0.001), *PPARγ2* (*F*(2,53) = 3.7, *p* < 0.05) as well as for adiponectin (*F*(2,52) = 10.8, *p* < 0.001), when compared with control mice (Figure 4).

### Subordination but not dominance stress exacerbated molecular signatures of MetS and insulin resistance in presence of high-fat diet

Subordinate mice fed a HFD-manifested glucose intolerance and insulin resistance (Sanghez et al., 2013) (Supplementary Table S1). Based on the results obtained in mice fed a standard diet we hypothesized that social subordination would further exacerbate the metabolic pattern of insulin resistance in the context of HFD feeding. At the single gene level we observed in subordinate mice fed a HFD, when compared to all other groups, a further significant decrease in expression in liver of genes for *IRS2* (*F*(3,68) = 11.3, *p* < 0.0001) and *CpT1α* (*F*(3,68) = 27.9, *p* < 0.0001; Figure 2B), and decreased expression in skeletal muscle of genes for *IRS2* (*F*(3,68) = 10.5, *p* < 0.0001) and *PPARγ1* (*F*(3,65) = 3.5, *p* < 0.05; Figure 3B), and decreased expression in pWAT of genes for *PPARγ1* (Figure 4B; *F*(3,66) = 8.4, *p* < 0.0001). Subordinate mice fed HFD also showed a downregulation in liver of expression of *PEPCK* and pyruvate carboxylase (*PC*) (Figure 2B; *F*(3,68) = 3.9, *p* < 0.05, *F*(3,68) =  3.38, *p* < 0.05, respectively), whereby excluding a significant role of gluconeogenesis on reported fasting hyperglycemia (Sanghez et al., 2013). In agreement with the relatively conserved homeostasis in dominant mice-fed HFD, this group only showed minor molecular changes (Figures 2–4).

### Pathway analysis reveals minor global metabolic changes in control mice fed a high-fat diet

The pathway analysis conducted in the pWAT of control mice fed with HFD revealed an upregulation of glucose uptake, growth and proliferation sub-pathways downstream of leptin in the adipokines pathway, and a downregulation in the nodes involved in antilipolysis in the insulin pathway compared to control mice fed standard diet (Supplementary Figure S1). Overall, the insulin and *PPAR* pathways in muscle and liver remained substantially unaffected after three weeks of HFD (Supplementary Figure S1). Finally, protein synthesis, proliferation and differentiation sub-pathways in the insulin pathways were upregulated in skeletal muscle (Supplementary Figure S1). Overall, short-term HFD was sufficient to induce mild transcriptional alterations in insulin-resistance biomarkers in metabolic tissues.

### Opposite effect of social status on the molecular signature of MetS and insulin resistance in mice fed standard diet

**Figure 5.**
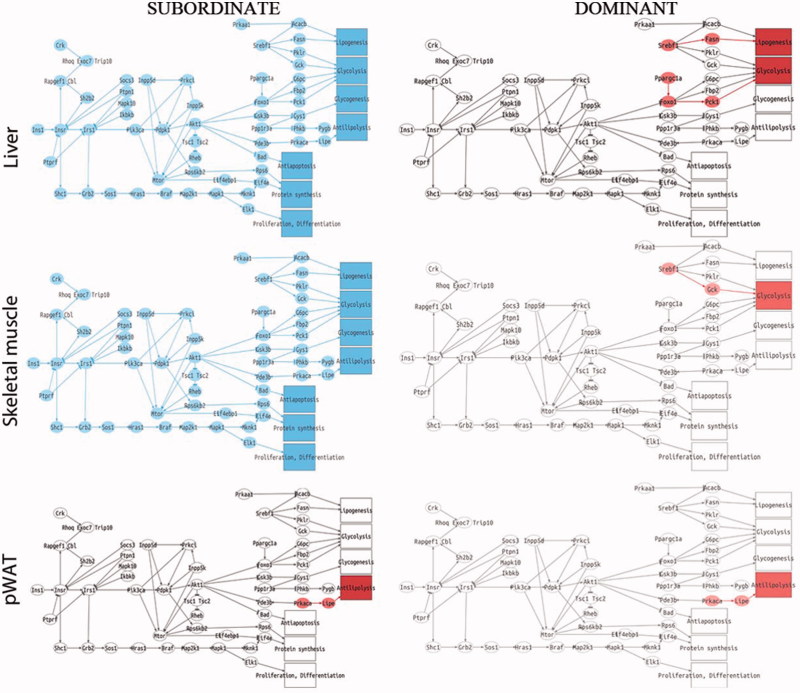
Insulin signaling pathway analysis in subordinate (SUB) and dominant (DOM) mice fed standard diet compared to controls (CON), based on PCR data from tissues shown in Figures 2–4. Representation of insulin pathway in liver, skeletal muscle, and pWAT. Sub-pathways significantly upregulated in the condition of interest (SUB or DOM) compared to CON are represented with all this group of nodes in red. Sub-pathways significantly downregulated are represented with all the nodes in blue. SUB mice showed a downregulation in all the sub-pathways in the insulin pathway for both liver and muscle. In contrast DOM showed only an upregulation of glycolysis in skeletal muscle, and glycolysis and lipogenesis in liver. Both SUB and DOM mice showed an upregulated antilipolysis sub-pathway in pWAT. Further details on the representation of the pathway analysis are in Figure S4. This figure will be best described with colors at the online version.

**Figure 6.**
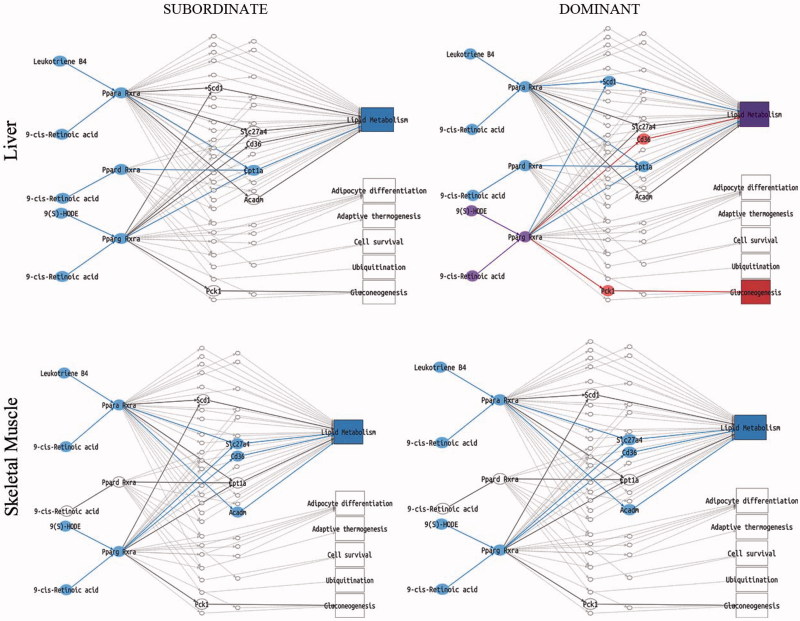
PPARs signaling pathway analysis in subordinate (SUB) and dominant (DOM) mice fed standard diet compared to controls (CON). Representation of PPARs pathway in liver and skeletal muscle, based on PCR data from tissues shown in Figures 2–4. Sub-pathways significantly upregulated in the condition of interest (SUB or DOM) compared to CON are represented with all the nodes in red. Sub-pathways significantly downregulated are represented with all the nodes in blue. SUB mice showed a downregulation in the lipid metabolism sub-pathway in liver and skeletal muscle when compared to CON. DOM exhibited a downregulation of lipid metabolism in skeletal muscle while in liver the lipid metabolism node is represented with a purple color identifying a node belonging to more than one significant sub-pathway having different behaviors (CD36-upregulated and Sc1-downregulated). DOM also showed an upregulation of gluconeogenesis in the liver. Further details on the representation of the pathway analysis appear in the Figure 1 legend. Further details on the representation of the pathway analysis appear in Figure S5. This figure will be best described with colors at the online version.

**Figure 7.**
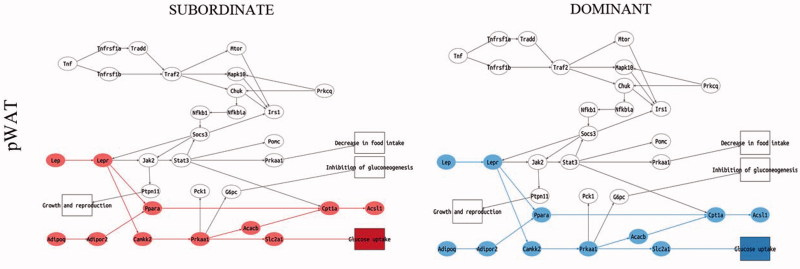
Adipokine signaling pathway analysis in the pWAT in subordinate (SUB) and dominant (DOM) mice fed standard diet compared to controls (CON), based on PCR data from tissues shown in Figures 2–4. Sub-pathways significantly upregulated in the condition of interest (SUB or DOM) compared to CON are represented with all this group of nodes in red. Sub-pathways significantly downregulated are represented with all the nodes in blue. SUB showed an upregulation in glucose uptake an adiponectin sub-pathways while DOM showed a downregulation compared to CON. Further details on the representation of the pathway analysis appear in Figure S3. This figure will be best described with colors at the online version.

We next performed a pathway analysis on the liver, skeletal muscle, and pWAT of subordinate and dominant mice fed standard diet or HFD compared to respective control groups. The liver and skeletal muscle of subordinate mice fed standard diet manifested a significant downregulation of all sub-pathways in the insulin pathway (Figure 5) and lipid metabolism in the PPAR signaling pathway (Figure 6). Conversely, in the pWAT, we observed an upregulation of the nodes immediately downstream of antilipolysis in the insulin pathway (Figure 5) as well as an upregulation of the leptin sub-pathway with all the nodes involved in glucose uptake and FFA metabolism (Figure 7). Moreover, the pathway downstream of adiponectin was upregulated in pWAT (Figure 7). Overall, we conclude that at this early stage of development of metabolic disorder, subordinate mice manifest molecular signatures of insulin resistance in liver and muscle while the adipose tissue remained insulin sensitive, though showing an increase in pathways facilitating fat accrual. Remarkably, the worsening of insulin resistance associated with being a subordinate is larger in magnitude compared to the effects of HFD in control mice (Figure S1).

In agreement with the healthy metabolic phenotype (Supplementary Table S1), the gene expression and pathway analysis showed a substantially normal profile in metabolic tissue from dominant mice fed standard diet. Notably, dominant mice showed upregulated sub-pathways for glycolysis and lipogenesis in the insulin pathway and downregulated leptin and adiponectin sub-pathways in the main adipokines pathway in pWAT (Figure 5); in addition the gluconeogenesis sub-pathway in the PPARs pathway in the liver was upregulated (Figure 6).

Finally, we performed a pathway analysis in tissue from subordinate and dominant mice fed a HFD and compared to control mice-fed HFD. Overall, the pathway analysis in both subordinate and dominant mice revealed only minor changes compared to the effects shown when fed standard diet, indicating that the global gene programing is more sensitive to subordination stress than to HFD per se (Supplementary Figure S2).

## Discussion

**Figure 8.**
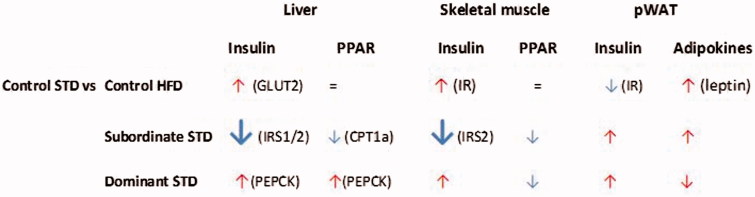
Summary of the main findings of the study. The relative size of arrows is proportional to the number of sub-pathways being significantly upregulated or downregulated within each pathway compared to controls. Color coding is congruent with the pathway analysis, i.e. red for upregulated and blue for downregulated pathways. When a candidate gene was associated and suggested to play a main role in the signaling pathway, its name is noted in parenthesis. STD, standard diet; HFD, high-fat diet; pWAT, perigonadal white adipose tissue. This figure will be best described with colors at the online version.

Obesity is a major risk factor for insulin resistance and T2D (Buettner et al., 2006; Karasawa et al., 2009; Surwit et al., 1988; Winzell & Ahrén, 2004). Similarly, psychosocial stress-induced metabolic disorders have been established in humans (Bose et al., 2009; Dallman et al., 2006), primates (Shively et al., 2009), and rodents (Bartolomucci et al., 2004,2009; Coccurello et al., 2009; Finger et al., 2012; Kuo et al., 2007). We developed a naturalistic model of CPS in which low social-rank mice (subordinate) are vulnerable to obesity and the metabolic-like syndrome while high social-rank mice (dominant) exhibit a healthy metabolic phenotype (Bartolomucci et al., 2009; Sanghez et al., 2013). In the present study, we tested the hypothesis that the opposite metabolic phenotype of subordinate and dominant mice is associated with changes in functional pathways relevant for insulin sensitivity, and glucose and lipid homeostasis. Our results demonstrate that subordinate mice manifest a molecular signature of insulin resistance in skeletal muscle and liver which is larger in magnitude than the effect of HFD per se in control mice and can be observed before hyperglycemia develops (Figure 8). Importantly, subordination stress interacted with HFD to exacerbate glucose intolerance and insulin resistance (Sanghez et al., 2013) as well as molecular changes in metabolically relevant organs. On the contrary, dominant mice showed an overall healthy metabolic phenotype (Sanghez et al., 2013) and largely normal expression of genes implicated in insulin sensitivity and glucose and lipid homeostasis.

In this study, we combined the quantitative PCR expression analysis of candidate genes and bioinformatic models based on the well-known descriptions of signaling pathways taken from the KEGG repository. KEGG devotes a complete section to cell signaling (Environmental Information Processing) that includes more than 40 pathways of signal transduction as well as signaling molecules and interactions. Within these pathways, the different sub-pathways leading from a specific stimulus received by a receptor protein to a particular response triggered by an effector protein have been defined (Sebastián-Leon et al., 2013, 2014). While the pathway models have been demonstrated to be quite accurate in terms of having a much reduced rate of false-positive and false-negative results, pathway models do have several weaknesses as well. One limitation is the fact that gene expression measurements are taken as proxies for protein production and subsequent activation. To limit this potential confounding factor we considered here the coordinated activation/deactivation of groups of genes within the context of a signaling sub-pathway. Furthermore, we have demonstrated that available phosphoproteomic measurements agree with the sub-pathway activity predictions made on the basis of the corresponding gene expression values (Amadoz et al., 2015). A second limitation of the method is that it can only be applied to known pathways. If, for some reason, the pathway underwent some modification, the model will ignore it and predictions will not be (to full extent) representative of the real signaling. This criticism, however, is extendable to any method based on predefined pathways and additionally, in order to minimize this risk, we selected genes with a rather well-established functional role.

### Severe downregulation of insulin signaling in liver and muscle in subordinate mice

Our previous work established that chronic subordination stress is associated with hyperphagia and dyslipidemia but normal glucose tolerance in mice fed standard diet (Sanghez et al., 2013). Conversely the development of glucose intolerance and insulin resistance required the additive effect of HFD and subordination stress (Bartolomucci et al., 2009; Dadomo et al., 2011; Sanghez et al., 2013). Here we showed that subordinate mice fed standard diet are already characterized by a marked downregulation of key genes implicated in glucose homeostasis as well as a global downregulation of the insulin signaling pathway in liver and muscle, but not the perigonadal adipose tissue, which is in agreement with the preferential deposition of fat in WAT (Figure 5). Among the metabolic and endocrine changes observed in subordinate mice, glucocorticoids and FFA can be considered the major factors contributing to the downregulation of the insulin signaling pathway (Kahn, 1994; Sanghez et al., 2013; Shpilberg et al., 2012; Stumvoll et al., 2005; Taniguchi et al., 2006). In particular, excessive FFA leading to lipotoxicity is a recognized risk factor for the development of insulin resistance (Virtue & Vidal-Puig, 2010) and has been mechanistically linked to a downregulation of the insulin and PPARs signaling pathway in metabolic tissues (Forman et al., 1996; Lewis et al., 2002). Interestingly, *CPT1α*, which is essential for fatty acid oxidation (Sebastián et al., 2009), was downregulated in muscle and liver of subordinate mice. *CPT1α* inhibition reduces long-chain fatty acid (LCFA) transport into and oxidation in muscle mitochondria resulting in an increase in FFA levels (McGarry, 2002), thus potentially contributing to the dyslipidemia observed in our model (Sanghez et al., 2013). Decreased *IRS2* appears to be the key molecular node critically downregulated in the signaling pathway of subordinate mice (Taniguchi et al., 2006; Figures 2 and 3). Germline or conditional ablation of *IRS2* as well as viral delivery of antisense oligonucleotides for *IRS2* leads to T2D in mice (Taniguchi et al., 2006; Withers et al., 1998). Conversely, pharmacologically induced upregulation of *IRS2* leads to improved glucose tolerance (Cao et al., 2011). Altogether, the changes in gene expression were observed in the context of euglycemia and increased *GLUT2* and *IR* in the liver, thus suggestive of the development of compensatory mechanisms able to normalize circulating glucose (Guillam et al., 1997; Thorens et al., 2000).

Molecular changes observed in subordinate mice fed with standard diet (as well as those fed HFD) were larger in magnitude compared to the effects exerted by a comparable HFD treatment in control mice. This suggests that downregulation of the insulin signaling pathway should be regarded as an early molecular biomarker of T2D (Sanghez et al., 2013). Several lines of evidence support this conclusion. First, human studies of T2D showed that insulin sensitivity and glucose disposal are defective in still normoglycaemic patients more than a decade before diagnosis of the disease. Second, reduced insulin signaling has been reported for insulin-resistant and diabetic patients as well as in most of the animal models of T2D (Olefsky et al., 1982). Third, in contrast to IRS1/2 deficient strains, mice heterozygous for *IRS1* showed hyperinsulinemia and glucose intolerance only in the presence of obesity (Shirakami et al., 2002). Finally, mice heterozygous for double *IR* and *IRS1* gene deficiency (with a ∼50–70% reduction in the level of protein expression and function) develop a slow onset T2D (Brüning et al., 1997). Interestingly, only a subgroup of double heterozygous mice developed T2D and the causal factor remains unexplained. In this scenario, our data suggest that chronic stress might be a relevant environmental factor explaining individual vulnerability to develop T2D. Specifically, our model supports the multistage and polygenic model for T2D and suggests that the disease develops in the presence of concomitant environmental/genetic risk factors. In support, we recently showed that subordination stress aggravates glucose intolerance in leptin receptor mutant db/db mice (Razzoli et al., 2015a).

### Dominant mice are characterized by a substantially normal expression of molecular markers of insulin resistance in metabolic tissues

We previously established that dominant mice in the CPS model manifest a healthy metabolic phenotype characterized by normal body weight, lipid profile and glucose tolerance despite the mice being hyperphagic and showing sustained stress-induced hyperactivation of the hypothalamo–pituitary–adrenal (HPA) axis (Sanghez et al., 2013). In support, using a combined candidate gene and pathway analysis, we demonstrated here that dominant mice have a substantially normal expression of molecular markers of insulin resistance in metabolic tissues. The more prominent phenotypes of dominant mice are sustained increase in body temperature, increased energy expenditure, hyperactivity, and increased sympathetic tone to adipose tissue, resulting in smaller adipocyte diameter (Bartolomucci et al., 2004,2009; Moles et al., 2006). Additionally, preventing stress-induced hyperphagia with a pair-feeding protocol normalizes weight gain and obesity, and improves glucose intolerance in subordinate mice (Razzoli et al., 2015b) while dominant mice showed a substantial weight and fat mass loss (Sanghez et al., unpublished). Overall, the more likely explanation for the healthy metabolic phenotype shown by dominant mice is that the high energetic cost in establishing and maintaining dominance (Moles et al., 2006; Sapolsky, 2005) prevents the development of dyslipidemia and lipotoxicity observed in subordinate mice, thus limiting the development of glucose intolerance even in the presence of hyperphagia and HFD feeding. Sympathetic hyperactivity can be associated with severe disease such as heart failure and atherosclerosis (Fisher et al., 2009). Accordingly, it remains to be investigated whether dominant mice develop other stress-associated diseases despite being apparently metabolically healthy. This conclusion is supported by accumulating evidence for a physiological “cost of being dominant” in naturalistic and semi-naturalistic settings (Bartolomucci et al., 2005; Gesquiere et al., 2011; Sapolsky, 2005). A limitation of the present study is that the metabolic and molecular analysis was conducted in healthy wild-type mice only. The observed metabolic effects suggest that genetic/disease mouse models showing impaired insulin signaling should be more vulnerable to stress-induced T2D, while mice carrying a transgenic over-expression of IRS1/2 in liver and or muscle should be protected from the metabolic derangement of subordination stress. Further studies are required to test this mechanistic hypothesis.

## Conclusions

In summary, we have demonstrated that three weeks of CPS (in mice housed at room temperature (Razzoli et al., 2016)) resulted in a molecular signature of insulin resistance in liver and muscle but not in the pWAT of subordinate mice. Remarkably, molecular signatures of insulin resistance in subordinate mice fed a standard diet were larger in magnitude compared to changes in control mice on a HFD. Conversely, dominance status conferred a protection against stress-induced molecular derangements that lead to T2D diabetes.

It is appropriate to point out that nongenetic mouse models developed so far have largely failed to recapitulate the complex metabolic disorder observed under conditions of chronic stress characterized by insulin resistance observed in the human clinical population (Harris, 2015). Here, we established a model of physiological and molecular signature of insulin resistance in an outbred mouse strain characterized by high genetic heterogeneity (Aldinger et al., 2009), thus extending the generalizability of the results obtained to a general human population.

## Notes

**Supplementary material available online** Supplementary Figures S1 and S2 Supplementary Tables S1 and S2
